# A reference genome assembly of the alpine forage grass *Elymus nutans*

**DOI:** 10.1111/pbi.70117

**Published:** 2025-06-18

**Authors:** Dan Chang, Shangang Jia, Ming Sun, Tao Huang, Huanhuan Lu, Jiajun Yan, Changbing Zhang, Minghong You, Jianbo Zhang, Lijun Yan, Wenlong Gou, Xiong Lei, Xiaofei Ji, Yingzhu Li, Decai Mao, Qi Wu, Ping Li, Hongkun Zheng, Xiao Ma, Xuebin Yan, Quanlan Liu, Xiaofan He, Wengang Xie, Daxu Li, Shiqie Bai

**Affiliations:** ^1^ Sichuan Academy of Grassland Sciences Chengdu China; ^2^ College of Life Sciences and Agri‐forestry Southwest University of Science and Technology Mianyang China; ^3^ College of Grassland Science and Technology China Agricultural University Beijing China; ^4^ Biomarker Technologies Corporation Beijing China; ^5^ College of Pastoral Agriculture Science and Technology Lanzhou University Lanzhou China; ^6^ College of Animal Science, Guizhou University Guiyang China; ^7^ College of Grassland Science and Technology, Sichuan Agricultural University Chengdu China; ^8^ College of Animal Science and Technology, Yangzhou University Yangzhou China; ^9^ College of Biological Engineering, Qingdao University of Science & Technology Qingdao China; ^10^ College of Grassland Science, Gansu Agricultural University Lanzhou China

**Keywords:** *Elymus nutans*, gemome assembly, Tibet plateau, Triticeae forage grass, Y subgenome

Dear Editor,

*Elymus nutans* Griseb. (Poaceae: Triticeae, 2n = 6x = 42) is a dominant perennial plant species (Figure 1a) in the Qinghai‐Tibetan Plateau in China (Liu *et al*., 2022), where it serves as an important forage grass with high yields, high nutritional value and good palatability for herbivorous ruminant animals.

**Figure 1:**
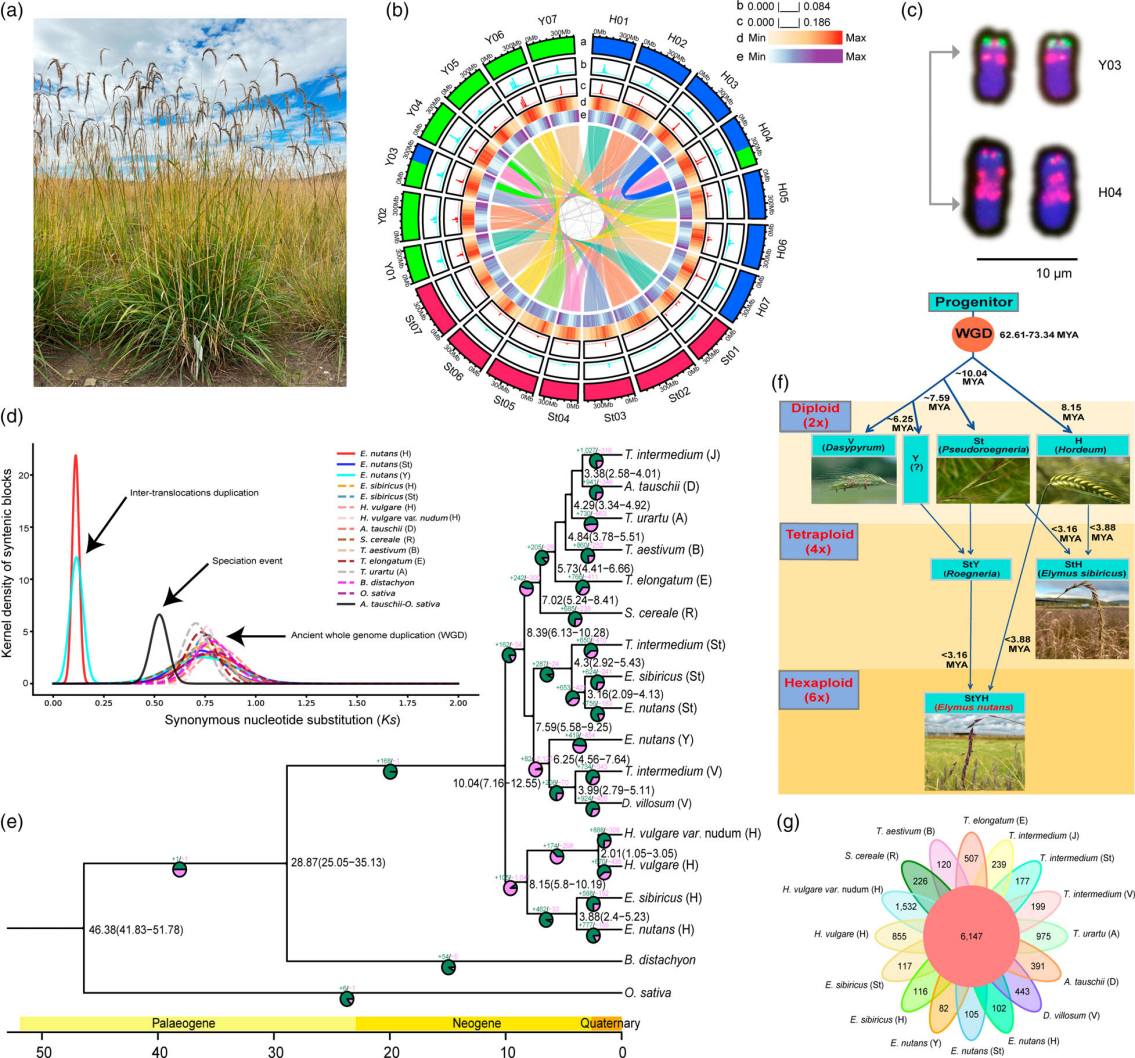
The reference genome of *Elymus nutans* provides insights into genome evolution and adaptation to the Qinghai‐Tibetan Plateau. (a) *E. nutans* cv. Aba in the Tibetan Plateau. (b) Overview of the genomic features of the *E. nutans* genome. Layer a, chromosome ideograms; layer b, density of wheat LTR retrotransposons Cereba/Quinta; layer c, density of maize centromeric satellites; layer d, TE density; layer e, high‐confidence gene density. (c) Translocated fragments identified by FISH, with green signals from H subgenome. (d) Ks distribution for whole‐genome duplication (WGD) events in *E. nutans* and other selected species. (e) Divergence time (based on TimeTree) and gene families for expansion (green) and contraction (red), in phylogenetic tree of 12 related species. (f) Model for genome evolution of *E. nutans*. (g) Venn diagram of gene families among subgenomes in Triticeae family members.

The genome size of *E. nutans* is estimated based on flow cytometry and k‐mer analysis, respectively (Figure S1). Using advanced sequencing technology, we generated an allohexaploid reference genome for *E. nutans*, representing the three sets of chromosomes (subgenomes St, Y and H). Initial contigs were assembled from long reads obtained using Oxford Nanopore Technology (ONT, 133.86×, N50 > 29 kb; Table S1), which were polished based on Illumina short reads (Table S2). We assembled the contigs into 21 pseudo‐chromosomes using Hi‐C data (119.2×, Table S2). After data cleaning and error correction, we obtained a final genome assembly of 9.46 Gb with a contig N50 of 3.01 Mb consisting of 21 chromosomes. The total length of scaffolds is 3.27 Gb, 3.27 Gb and 2.83 Gb for H, St and Y subgenomes, respectively (Table S3). The chromosomes were further grouped into three subgenomes (StStYYHH) based on similarity to the genomes of barley (*Hordeum vulgare*; HH) and *Elymus sibiricus* (StStHH) (Figure 1b).

The benchmarking universal single‐copy orthologs (BUSCO) score of the *E. nutans* assembly is 96.6% and the long terminal repeats (LTR) assembly index (LAI) is 16.54, 14.87 and 17.20 for the St, Y and H subgenomes, respectively, confirming a high quality. We successfully mapped 99.64% ONT and 97.1% Illumina reads to the genome assembly and the uniform coverage of mapped reads showed the reliability of the assembly, which was supported by the Hi‐C heatmap. Synteny analysis revealed conservation among the three subgenomes, with one large reciprocal translocation detected between chromosomes H04 (175.1 Mb) and Y03 (153.8 Mb) (Figure 1b; Figure S2). This reciprocal translocation, which was further confirmed by fluorescence in situ hybridization (FISH) imaging using unique probes for subgenome H, is localized at one end of chromosome Y03 (Figure 1c). Collinearity between H04 and H03/St03 and between Y03 and Y04/St04 indicated the results from reciprocal translocation (Figure 1b). The syntenic blocks among the three subgenomes of E. nutans (St, Y and H), Xa, H, V, Y, St, R, E, B, A, D and J subgenomes in other Triticeae species also suggest a reliable assembly of the E. nutans genome and potential structural variations (Figure S3).

Among the *E. nutans* genome, 83.89% are annotated as repetitive sequences (Table S4) and up to 61.67% are grouped as LTRs and dominated by the most abundant LTRs of Copia and Gypsy (Table S4). Gene annotation based on de novo, homology and transcript‐based predictions resulted in 114 214 gene models, including 39 341, 40 837 and 33 541 gene models for subgenomes H, St and Y, with average gene lengths of 3392.60 bp, 3462.82 bp and 3409.88 bp, respectively (Table S5).

We determined the potential locations of centromeric regions in the assembly based on enrichment of the known centromeric sequences in wheat and maize (Figure 1b). The LTR retrotransposons Cereba/Quinta (GenBank accession no. FN564437.1) and the whole centromeric sequences were retrieved from the centromeres of wheat (NCBI accession no. GCA_022117705.1) and maize (Chen *et al*., 2023), respectively, and their alignments to the assembly pointed to the same locations with substantial overlap across all 21 chromosomes of the three subgenomes (Figure 1b; Table S6). The potential centromeric regions are in accordance with the enrichment of transposable elements (TEs) and the gene‐poor centromeric and pericentromeric regions (Figure 1b). We further observed the highest proportion of tandem repeats among the potential centromeric regions of the H, St and Y subgenomes, accounting for 26.55%, 19.41% and 21.11%, respectively (Table S7). However, these repeat units and their contents would like to be further confirmed in the future.

We explored the divergence of the three *E. nutans* subgenomes via sequence similarities with phylogenetically closely related species. The sequence identity in these species reached approximately 97.5% for subgenomes H and St (Figure S4). Similar to other Gramineae species, the distribution of Ks values formed peaks at 0.7–0.82 (Figure 1d), indicating that an ancient WGD event affecting the three subgenomes occurred approximately 62.61–73.34 million years ago (MYA). From a phylogenetic tree reconstructed using 18 subgenomes of 12 species (Figure 1e), we estimated the divergence time of the three subgenomes to be approximately 10.04 MYA, with Y and St further splitting ~7.59 MYA. Using divergence times and evolutionary relationships, we reconstructed a model for the evolutionary history of *E. nutans*, and it showed that hexaploid *E. nutans* (StStYYHH) occurred <3.16 MYA after the split of St subgenomes between *E. nutans* and *E. sibiricus* (Figure 1e,f) (Chen *et al*., 2024). The hybridization of an ancient diploid species (HH, e.g., *Hordeum*) and a tetraploid species (StStYY, e.g., *Roegneria*) (Figure 1f), rather than the one between StStHH and YY, is strongly supported by the facts that no diploid species (YY) are currently found in the world, and multiple hexaploidy species (StStYYWW, StStYYPP and StStYYHH) occurred as frequent events (Chen *et al*., 2024; Fan *et al*., 2013). The history of the Y subgenome could be traced to 6.25 MYA, when genome V in *Thinopyrum intermedium* and *Dasypyrum villosum* diverged from the ancestor of Y and V genomes (Figure 1e).

Gene family analysis in *E. nutans* and nine other Triticeae subgenomes identified 102, 105 and 82 gene families unique to *E. nutans* subgenomes H, St and Y, respectively, and 6147 gene families shared among subgenomes (Figure 1g). Expanded gene families were identified in the three subgenomes (Figure 1e), and enriched in pathways related to environmental adaptation (Figure S5), for example, strong UV‐B and drought stress in Tibetan Plateau. We collected and planted five lines of wild resources from different altitudes and locations (Table S8), conducted the transcriptomic studies under the treatments of UV‐B and drought stress, and performed the data validation by qRT‐PCR (Tables S9–S11). Weighted gene co‐expression network analysis (WGCNA) revealed that the DEGs under both UV‐B (black module) and drought stress (purple module) are highly enriched in glutathione transferase activity (Figure S6). We found the allohexaploid *E. nutans* genome harbours 342 *GST* genes (nine subfamilies), surpassing other species. Tau and phi subfamilies dominate, with *E. nutans*' St and H subgenomes showing exceptionally high tau member counts compared to wheat's subgenomes (Figure S7a; Table S12). Furthermore, we discovered the transcriptional responses of five phi and tau subfamily members (EVM0015335, EVM0002076, EVM0134842, EVM0087283 and EVM0141011) to the treatments of both drought and UV‐B, and their expressions exhibited significant differences between the lines (NM037 vs QH009, SC020 vs NM035) (Figure S7b,c). The *WRKY* transcription factor *EVM0129376_WRKY* played a role as a hub gene in both the networks for the two WGCNA modules (Figure S7d,e). These findings suggest that the *GST* members might interact with transcription factors of WRKY (such as EVM0129376) and others, and participate in responses to drought and UV‐B stresses (Dixon *et al*., 2002; Jiang *et al*., 2017).

In summary, our high‐quality assembly of the three subgenomes of the Triticeae forage grass *E. nutans* provides critical insights into the evolutionary history of this species, and will serve as a valuable resource for future studies on its adaptation to the extreme environmental conditions of the Qinghai‐Tibetan Plateau.

## Funding

This work was supported by the Science & Technology Department of Sichuan Province (Grant No. 2021YFYZ0013‐2, 2019YFN0170 and 2023YFSY0012), the Sichuan Provincial Department of Agriculture and Rural Affairs (Grant No. SCCXTD‐2025‐16), the National Center of Pratacultural Technology Innovation (under preparation) (Grant No. CCPTZX2023W01) and the Sichuan Provincial Forestry and Grassland Administration (Grant No. CXTD2025005).

## Author contributions

S.B. conceived the project. W.X. and D.L. provided the financial support and participated in the supervision of the project. D.C., H.L., J.Y., C.Z., M.Y., J.Z., L.Y., W.G., X.L., X.J., Y.L., D.M., Q.W., X.C., J.T., H.Z. and P.L. contributed to plant sample collection, DNA/RNA preparation, library construction and sequencing. X.M., X.Y. and Q.L. assisted with data analysis. S.J. and T.H. performed genome assembly and annotation and comparative genomic analyses. X.H. performed the screening of centromeric repeats. T.H. and M.S. performed transcriptome analysis and analysis of the *GST* gene family. S.J., D.C. and M.S. wrote and revised the manuscript.

## Data avilability statement

The genome assembly (accession no. GWHFAJN00000000.1) and raw sequencing data generated in this study, comprising ONT data, Illumina data, Iso‐seq data, and ChIP‐seq data, can be found in the Genome Sequence Archive at the National Genomics Data Center (https://ngdc.cncb.ac.cn/) under BioProject accession number PRJCA028418.

## Supporting information


**Figures S1–S7** Supplemental figures.
**Tables S1–S12** Supplemental tables.
Supplemental materials and methods.
