# Supplementary material for: A reference genome assembly of the alpine forage grass Elymus nutans
Source: Plant Biotechnol J. 2025 Jun 18;23(9):3900–2. doi: 10.1111/pbi.70117 (PMC12392932; doi:10.1111/pbi.70117)
Supplement: Supplementary file 1 — Figures S1–S7 Supplemental figures. Tables S1–S12 Supplemental tables. Supplemental materials and methods. [file PBI-23-3900-s001.pdf]

Supplemental information

A reference genome assembly of the alpine forage grass *Elymus nutans*

Dan Chang, Shangang Jia, Ming Sun, Tao Huang, Huanhuan Lu, Jiajun Yan, Changbing Zhang, Minghong You, Jianbo Zhang, Lijun Yan, Wenlong Gou, Xiong Lei, Xiaofei Ji, Yingzhu Li, Decai Mao, Qi Wu, Ping Li, Hongkun Zheng, Xiao Ma, Xuebin Yan, Quanlan Liu, Xiaofan He, Wengang Xie, Daxu Li, and Shiqie Bai

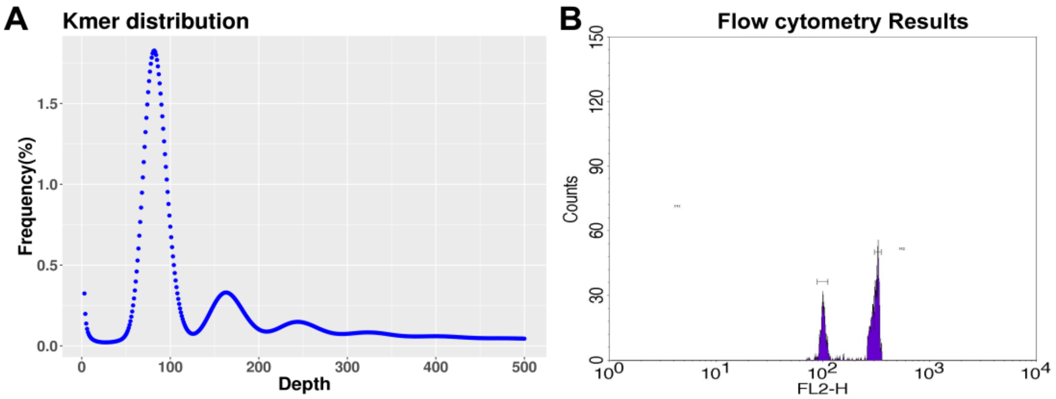

Supplemental Figure 1 Estimation of the genome size of *E. nutans*. (**A**) K-mer (k = 23) frequency distribution. The genome size was calculated using the formula: Genome size = K-mer number / peak depth, which resulted in an estimated genome size of 10.50 Gb. (**B**) Histograms showing G0/G1 nuclei peaks of *E. nutans* compared with *T. aestivum*. Based on the relative fluorescence intensity ratio, the ratio in genome sizes of *E. nutans* versus *T. aestivum* is 4.714. Therefore, the estimated size of the *E. nutans* genome is 10.84 Gb. The heterozygosity rate is 0.01%, and the repetitive sequence content reaches approximately 78.00%, indicating a high-repeat and large genome.

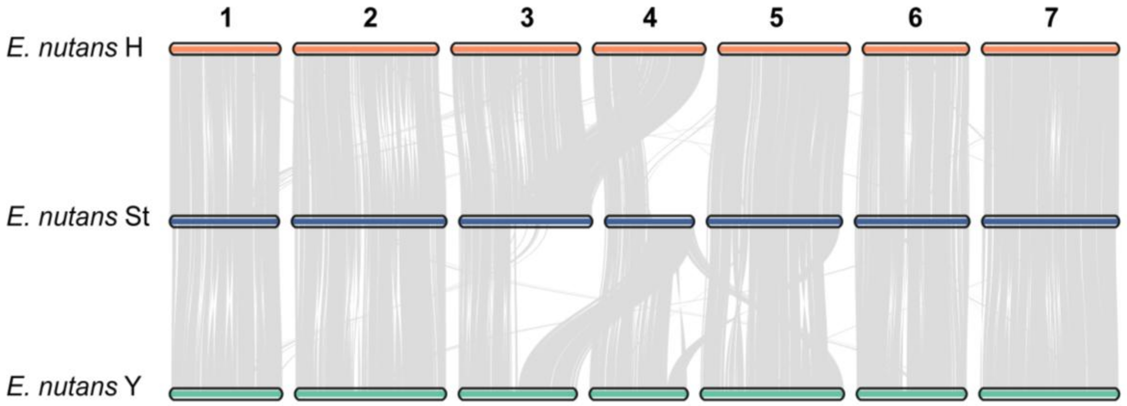

Supplemental Figure 2 Subgenome classification of *E. nutans* (*E. nutans* St, *E. nutans* H and *E. nutans* Y) and synteny analysis.

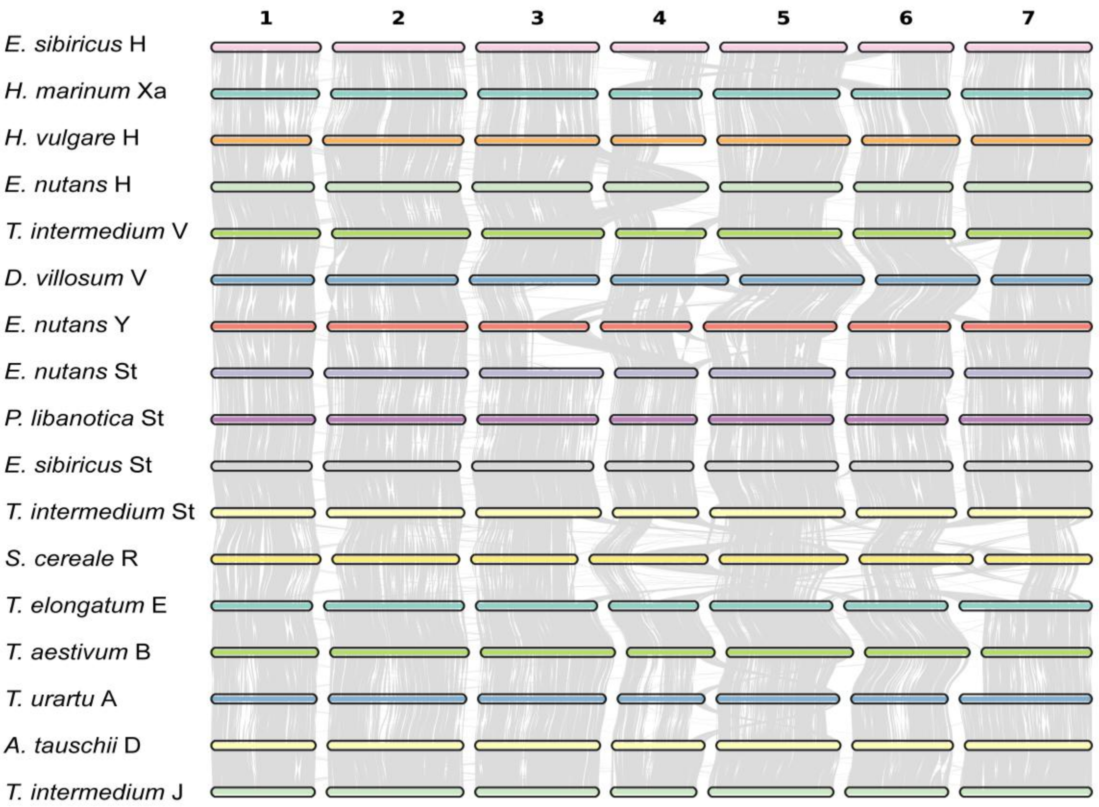

Supplemental Figure 3 Syntenic blocks among St, Y, H and A, B, D, E, J, R, V genomes of Triticeae species. The syntenic blocks suggest a reliable assembly of E. nutans genome, and potential structural variations.

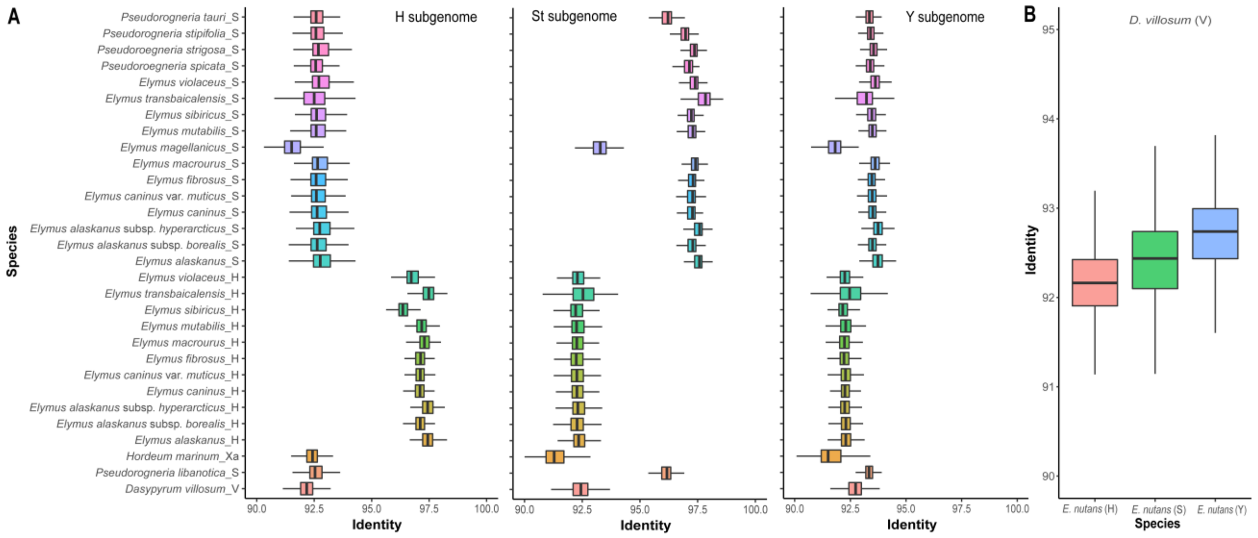

Supplemental Figure 4 Sequence similarities analysis. (**A**) Sequence similarities of reads from *Elymus* species, and diploid species *D. villosum* (V), *P. libanotica* (StSt), *H. vulgare* (HH), and *H. marinum* (XaXa) that were uniquely mapped to the H, St, and Y subgenomes of *E. nutans*. (**B**) Sequence similarities analysis of *D. villosum* (V) with the H, St, and Y subgenomes of *E. nutans*.

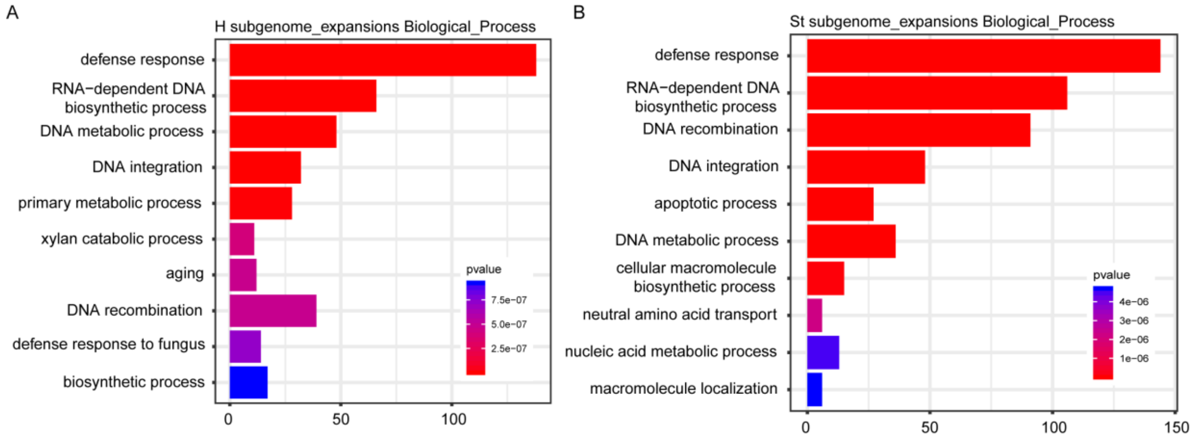

Supplemental Figure 5 Top 10 most significantly enriched GO biological process terms for the expanded gene families in the H (**A**) and St (**B**) subgenomes. The expanded gene families were significantly enriched in pathways related to environmental adaptation, with the conspicuous expansion of gene families associated with defense response processes in both H and St subgenomes.

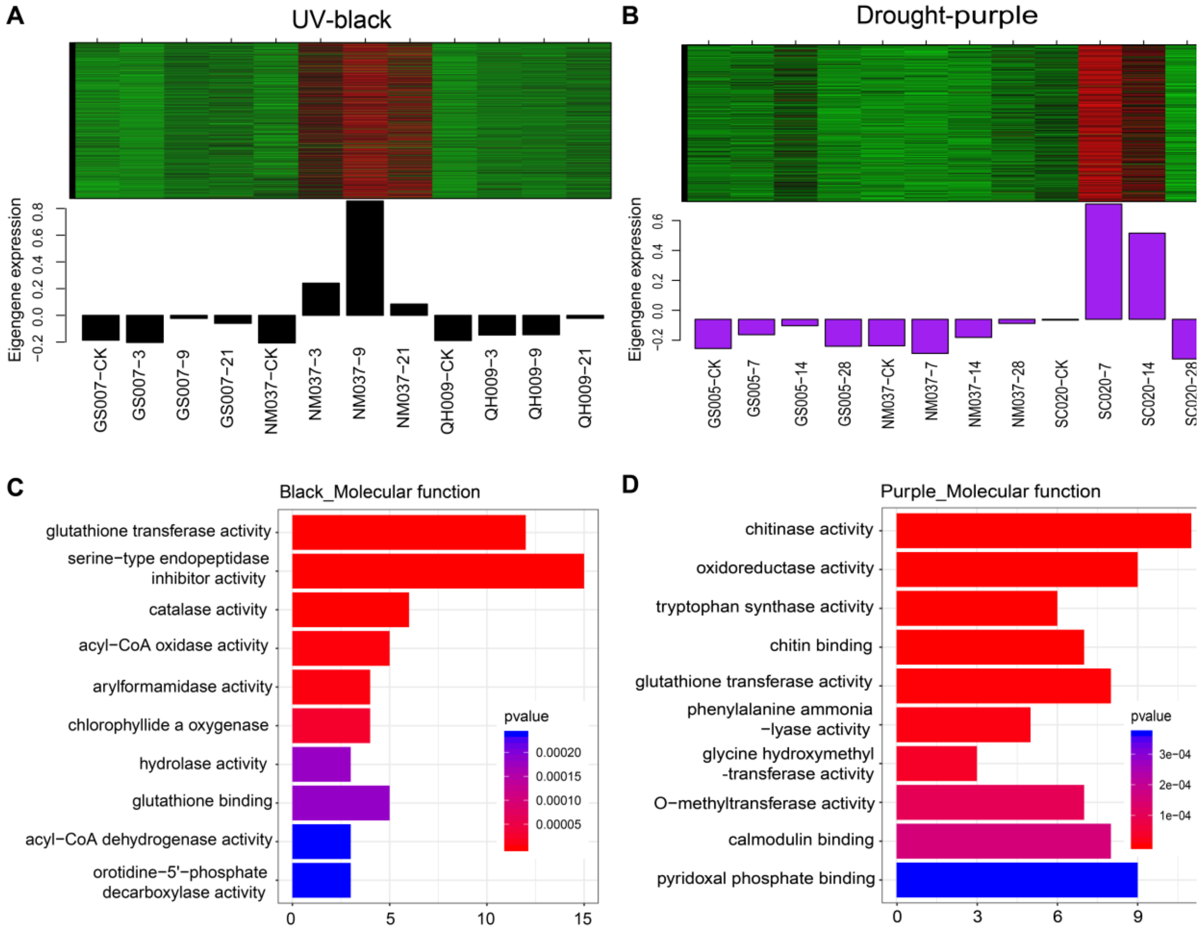

Supplemental Figure 6 Gene expression patterns and GO enrichment analysis of genes in the black and purple modules. (**A**) Gene expression patterns of genes in the black module detected by WGCNA following UV-B treatment. (**B**) Gene expression patterns of genes in the purple module detected by WGCNA following drought treatment. (**C**) Top 10 GO molecular function terms for the genes in the UV-B/black module. (**D**) Top 10 GO molecular function terms for the genes in the drought/purple module.

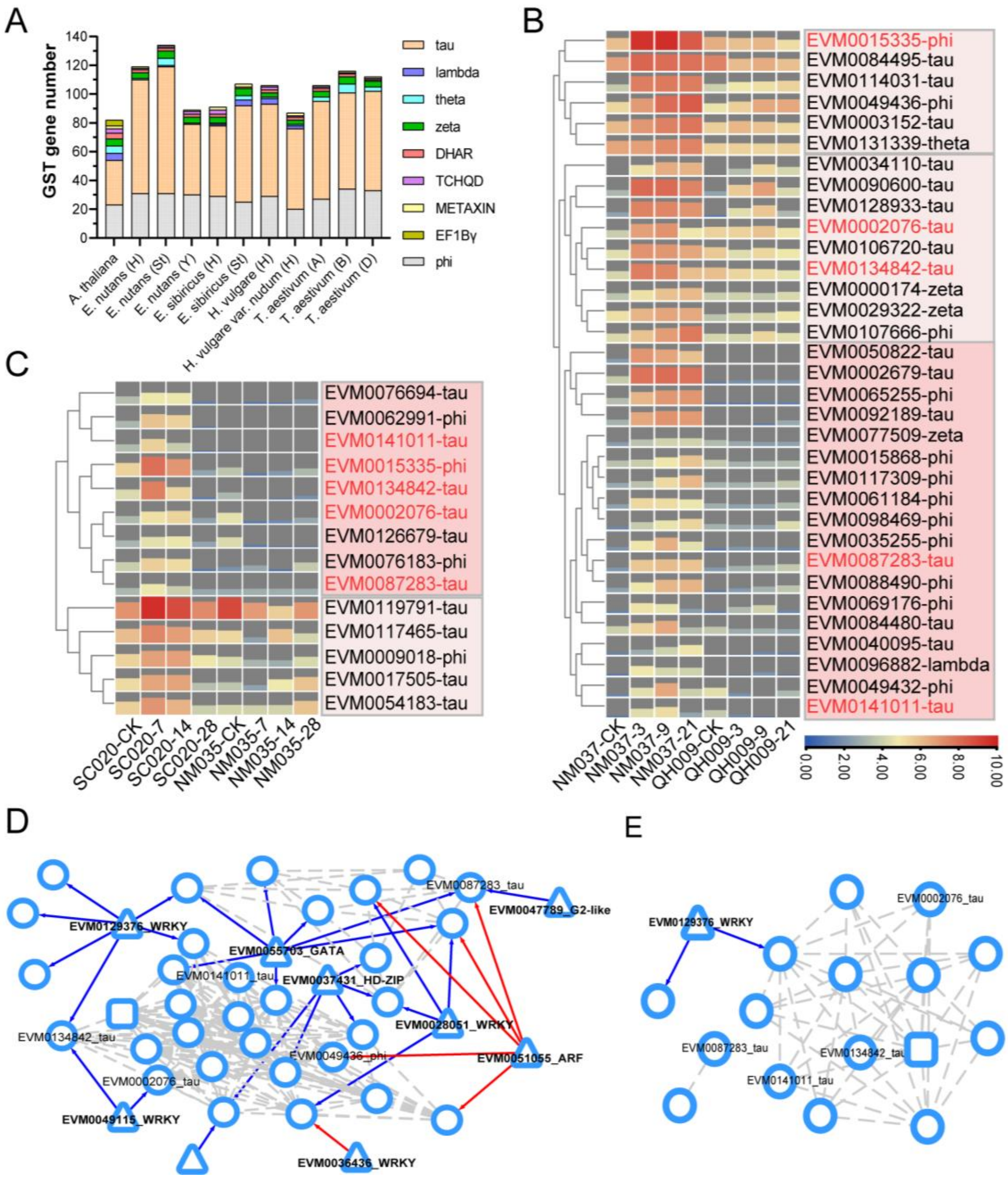

Supplemental Figure 7 GST genes participate in responses to drought and UV-B stresses. (**A**) *GST* genes in *E. nutans* compared with other plant species. (**B**) Expression patterns of *GST* genes in the WGCNA black module in tolerant (QH009) and sensitive (NM037) lines treated with UV-B. (**C**) Expression patterns of *GST* genes in the WGCNA purple module in tolerant (NM037) and sensitive (SC020) lines treated with drought. (**D**) Regulatory relationships between transcription factors and *GST* genes in the UV-B treatment experiment. (**E**) Regulatory relationships between transcription factors and *GST* genes in the drought treatment experiment. The co-expression networks in these two modules revealed strong interactions of WRKY, GATA, HD-ZIP, ARF, and G2-Like transcription factors with the five shared *GST* genes.

Supplemental Table 1 Summary of Nanopore sequencing data

Tissue	Total reads	Total bases	N50 (bp)	N90 (bp)	Mean length (bp)	Max length (bp)	Mean quality	BioProject accession number
Leaf	59,036,775	1,266,055,179,855	29,036	12,460	21,445	344,420	8.23	PRJCA028418

Supplemental Table 2 Summary of Illumina sequencing and Hi-C data

Data type	Tissue	Library	Total reads	Total bases (bp)	GC content (%)	Q30 (%)	BioProject accession number
Illumina sequencing data	Stem/Leaf/Root	T01	96,294,374	28,846,649,322	57.12	95.08	PRJCA028418
Hi-C data	Stem/Leaf/Root	H01	507,045,904	151,551,840,872	51.59	92.98	PRJCA028418
	Stem/Leaf/Root	H02	508,558,714	151,803,572,936	49.65	92.45	
	Stem/Leaf/Root	H03	517,750,260	154,561,581,828	49.95	92.81	
	Stem/Leaf/Root	H04	462,819,843	138,023,841,758	49.81	93.95	
	Stem/Leaf/Root	H05	457,810,106	136,552,117,232	49.82	93.30	
	Stem/Leaf/Root	H06	466,212,482	139,170,266,710	49.68	93.42	
	Stem/Leaf/Root	H07	621,009,962	185,159,884,998	49.68	92.90	

Supplemental Table 3 Assembly statistics of the *E. nutans* genome

	E*．*nutans	***E*．*nutans* H subgenome**	***E*．*nutans* St subgenome**	***E*．*nutans* Y subgenome**
**Chromosome statistics**				
ChrLen (bp)	8,865,216,234	3,103,342,108	3,056,091,254	2,705,782,872
Hi-C assembly ratio	94.69%	94.91%	93.54%	95.76%
**Scaffold statistics**				
ScfNum	10,740	1,275	1,951	1,153
ScfLen (bp)	9,458,615,163	3,269,857,642	3,267,036,055	2,825,641,198
**Contigs statistics**				
CtgNum	14,720	2,570	3,586	2,204
CtgN50 (bp)	3,009,349	3,309,141	2,426,056	3,857,394
CtgMax (bp)	33,078,935	33,078,935	16,109,970	25,828,108
GC	46.23%	46.17%	46.09%	46.41%

ChrLen: total length of chromosomes; Hi-C assembly ratio: the proportion of ordered contigs length to cluster contigs length; ScfNum: number of scaffolds; ScfLen: total length of scaffolds; CtgNum: number of contigs; CtgN50: contig N50; CtgMax: maximum sequence length of contigs; GC: GC content of genome.

Supplementary Table 4 Repetitive sequences in *E. nutans*

Repeat Class Description			*E. nutans*		*E. nutans* H subgenome		*E. nutans* St subgenome		*E. nutans* Y subgenome	
Repeat class	Type	Superfamily	bp	%	bp	%	bp	%	bp	%
Class I TEs	LTR	Copia	1,303,900,955	13.79	520,368,864	15.91	359,387,609	11	409,925,230	14.51
		Gypsy	3,937,238,995	41.63	1,350,333,150	41.3	1,390,746,252	42.57	1,173,640,216	41.54
		Unknown	591,340,798	6.25	241,523,820	7.39	213,458,654	6.53	131,609,961	4.66
	nonLTR	LINE	31,231,910	0.33	13,459,742	0.41	9,229,518	0.28	8,421,629	0.3
		SINE	813,401	0.01	119,480	0	349,688	0.01	341,246	0.01
Class II TEs	DNA_transposon		2,110,860	0.02	551,925	0.02	787,995	0.02	769,138	0.03
	TIR	CACTA	1,209,659,372	12.79	324,248,132	9.92	441,425,583	13.51	425,370,393	15.05
		Mutator	165,246,819	1.75	58,513,047	1.79	59,835,976	1.83	44,871,659	1.59
		PIF_Harbinger	106,833,082	1.13	40,352,727	1.23	35,227,984	1.08	30,899,207	1.09
		Tc1_Mariner	141,894,691	1.5	46,524,040	1.42	52,785,247	1.62	42,053,305	1.49
		hAT	37,967,324	0.4	13,403,624	0.41	13,996,666	0.43	10,404,995	0.37
		Unknown	12,359,774	0.13	4,090,763	0.13	4,247,328	0.13	3,986,806	0.14
	nonTIR	Helitron	303,451,477	3.21	105,843,646	3.24	113,067,128	3.46	83,268,857	2.95
Repeat regions			90,348,161	0.96	27,216,380	0.83	34,066,244	1.04	25,574,199	0.91
Total			7,934,397,619	83.89	2,746,549,340	84	2,728,611,872	83.52	2,391,136,841	84.63

Supplementary Table 5 Comparison of predicted gene models in *E. nutans* versus six additional species

	*E. nutans**	*E. nutans H*	*E. nutans St*	*E. nutans Y*	*Sorghum bicolor*	*Triticum aestivum*	*Zea mays*	*Arabidopsis thaliana*	*Hordeum vulgare*	*Oryza sativa*
**GeneNum**	114,214	39,341	40,837	33,541	34,129	107,544	35,615	27,381	39,718	38,852
**GeneLen**	390,341,448	133,468,086	141,411,111	114,370,622	126,726,290	375,144,978	157,589,684	60,368,916	238,572,893	110,901,512
**AveGenlen**	3,417.63	3,392.60	3,462.82	3,409.88	3,713.16	3,488.29	4,424.81	2,204.77	6,006.67	2,854.46
**ExonLen**	165,415,624	57,305,363	59,238,974	48,325,483	62,148,246	168,906,626	58,505,555	40,579,948	40,906,926	41,259,390
**AveExonLen**	1,448.30	1,456.63	1,450.62	1,440.79	1,820.98	1,570.58	1,642.72	1,482.05	1,029.93	1,061.96
**ExonNum**	528,136	180,165	187,495	158,794	166,869	502,038	170,559	145,401	154,975	160,012
**CDSLen**	145,823,238	50,520,939	52,161,198	42,647,313	39,640,380	133,312,040	41,331,000	33,343,421	40,906,926	41,259,390
**AveCDSlen**	1,276.75	1,284.18	1,277.30	1,271.50	1,161.49	1,239.60	1,160.49	1,217.76	1,029.93	1,061.96
**CDSNum**	514,468	175,478	182,131	155,193	153,976	477,350	160,069	140,295	154,975	160,012
**IntronLen**	224,925,824	76,162,723	82,172,137	66,045,139	64,578,044	206,238,352	99,084,129	19,788,968	197,665,967	69,642,122
**AveIntronLen**	1,969.34	1,935.96	2,012.20	1,969.09	1,892.18	1,917.71	2,782.09	722.73	4,976.74	1,792.50
**IntronNum**	413,922	140,824	146,658	125,253	132,740	394,494	134,944	118,020	115,257	121,160

GeneNum: number of genes; GeneLen: total gene length; AveGenelen: average gene length; ExonLen: total exon length; AveExonLen average exon length per gene; ExonNum: number of exons; CDSLen: total coding sequence (CDS) length; AveCDSlen: average CDS length per gene; CDSNum: number of CDSs; IntronLen: total intron length; AveIntronLen: average intron length per gene; IntronNum: number of introns. *The genes in *E. nutans* include the ones on the three subgenomes and unplaced contigs.

Supplementary Table 6 Centromeric coordinates of St, Y and H subgenomes in *E. nutans*

Chromosome	TE based centromeric regions			Satellites based centromeric regions			Combined centromeric regions		
	Start	End	Length	Start	End	Length	Start	End	Length
**H01**	124,000,001	140,000,000	16,000,000	129,000,001	131,000,000	2,000,000	124,000,001	140,000,000	16,000,000
**H02**	203,000,001	212,000,000	9,000,000	203,000,001	211,000,000	8,000,000	203,000,001	212,000,000	9,000,000
**H03**	188,000,001	197,000,000	9,000,000	188,000,001	202,000,000	14,000,000	188,000,001	202,000,000	14,000,000
**H04**	228,000,001	232,000,000	4,000,000	227,000,001	232,000,000	5,000,000	227,000,001	232,000,000	5,000,000
**H05**	136,000,001	142,000,000	6,000,000	137,000,001	154,000,000	17,000,000	136,000,001	154,000,000	18,000,000
**H06**	200,000,001	208,000,000	8,000,000	201,000,001	208,000,000	7,000,000	200,000,001	208,000,000	8,000,000
**H07**	226,000,001	240,000,000	14,000,000	231,000,001	241,000,000	10,000,000	226,000,001	241,000,000	15,000,000
**S01**	159,000,001	164,000,000	5,000,000	159,000,001	165,000,000	6,000,000	159,000,001	165,000,000	6,000,000
**S02**	242,000,001	246,000,000	4,000,000	242,000,001	244,000,000	2,000,000	242,000,001	246,000,000	4,000,000
**S03**	169,000,001	181,000,000	12,000,000	176,000,001	181,000,000	5,000,000	169,000,001	181,000,000	12,000,000
**S04**	151,000,001	158,000,000	7,000,000	152,000,001	158,000,000	6,000,000	151,000,001	158,000,000	7,000,000
**S05**	138,000,001	142,000,000	4,000,000	138,000,001	141,000,000	3,000,000	138,000,001	142,000,000	4,000,000
**S06**	194,000,001	200,000,000	6,000,000	195,000,001	198,000,000	3,000,000	194,000,001	200,000,000	6,000,000
**S07**	230,000,001	238,000,000	8,000,000	235,000,001	237,000,000	2,000,000	230,000,001	238,000,000	8,000,000
**Y01**	114,000,001	123,000,000	9,000,000	112,000,001	123,000,000	11,000,000	112,000,001	123,000,000	11,000,000
**Y02**	188,000,001	193,000,000	5,000,000	185,000,001	193,000,000	8,000,000	185,000,001	193,000,000	8,000,000
**Y03**	165,000,001	175,000,000	10,000,000	166,000,001	175,000,000	9,000,000	165,000,001	175,000,000	10,000,000
**Y04**	155,000,001	160,000,000	5,000,000	156,000,001	160,000,000	4,000,000	155,000,001	160,000,000	5,000,000
**Y05**	115,000,001	132,000,000	17,000,000	115,000,001	131,000,000	16,000,000	115,000,001	132,000,000	17,000,000
**Y06**	164,000,001	168,000,000	4,000,000	164,000,001	169,000,000	5,000,000	164,000,001	169,000,000	5,000,000
**Y07**	212,000,001	230,000,000	18,000,000	221,000,001	230,000,000	9,000,000	212,000,001	230,000,000	18,000,000

Supplementary Table 7 Tandem repeats identification for combined centromeric regions of St, Y and H subgenomes in *E. nutans*

Type	H subgenome		St subgenome		Y subgenome	
	Consensus sequence	Percentage (%)	Consensus sequence	Percentage (%)	Consensus sequence	Percentage (%)
Microsatellite	TCGTGTTCT	9.77	AT	4.22	TTC	4.92
	GAAGAACAC	5.24	GA	3.88	AGA	4.14
	GAACACGAA	4.50	AAAG	3.82	AAG	3.66
	AT	3.58	TC	3.75	AT	3.44
	AGA	3.45	AGA	3.75	GA	2.48
	——		——		TA	2.48
	All microsatellites	26.55	All microsatellites	19.41	All microsatellites	21.11
Minisatellite	AGGCCCCGGACATCCGGCCA	0.47	GAGGTCTTGATGATCCTATGATGGAGATCCTTCTCCCTAGA	0.22	CATGATCTCCATCAT	0.12
	GGCCGGACGTCCGGGGCCTT	0.34	ATCAAGACCTCTCTAGGGAGAAGGATCCCCATCATAGGATC	0.18	GGACTAGGATTGGTGGTGGTATAT	0.12
	AACAAAAAATCACAAG	0.29	CATTTCTTTTCAAGACCTCTCGAGGCGTCGCTTTTAGACACCCGTTTGG	0.18	TGGTATTGATGAAAC	0.11
	CCCGGACATCCGGCCCCTGG	0.25	TGATGATGATTTACTTGG	0.17	AGAGTAGCATCTTC	0.10
	CAGGGGCCGGACGTCCGGGC	0.24	ACTTCATCAAGTGCT	0.16	ATCTCCAAAGTCACCGGCATG	0.10
	All minisatellites	1.59	All microsatellites	0.92	All microsatellites	0.56
Satellite	All satellites	2.91	All satellites	1.40	All satellites	1.27

Microsatellite: Consensus size < 10; Minisatellite: 10 ≤ Consensus size < 100; Satellite: Consensus size ≥ 100. Classification of tandem repeat types based on Consensus size.

Supplementary Table 8 *E nutans* accessions subjected to RNA-seq following UV and drought treatments

Line no.	Geographical origin	Elevation (m)	Mean annual rainfall (mm)	UV-B intensity (MJ/m^2)
QH009	Chendu County, Gansu Province	4,310	——	874.23
GS007	Luqu County, Gansu Province	3,110	——	738.63
NM037	Xilingol League in Inner Mongolia autonomous Region	1,380	365.1	623.88
GS005	Xiahe County, Gansu Province	3,140	516	-
SC020	Junba, Sichuan province	3,563	722.2	-

Supplementary Table 9 RNA-seq data following UV-B treatment

Sample ID	Geographical origin	Tissue	Clean reads	Clean bases	GC (%)	Q30 (%)
GS007-CKa	Gansu, China	Leaf	22,714,901	6,787,594,308	55.88	94.85
GS007-CKb	Gansu, China	Leaf	25,220,920	7,527,816,656	55.21	94.61
GS007-CKc	Gansu, China	Leaf	27,137,450	8,107,116,566	55.17	94.66
GS007-3a	Gansu, China	Leaf	25,485,948	7,599,005,606	55.87	94.81
GS007-3b	Gansu, China	Leaf	23,492,696	7,022,486,848	55.99	94.85
GS007-3c	Gansu, China	Leaf	21,684,610	6,478,272,796	56.43	94.72
GS007-9a	Gansu, China	Leaf	21,901,042	6,545,327,778	55.44	94.77
GS007-9b	Gansu, China	Leaf	27,520,854	8,223,949,200	55.08	94.69
GS007-9c	Gansu, China	Leaf	22,040,144	6,585,174,946	54.39	94.57
GS007-21a	Gansu, China	Leaf	28,306,018	8,452,148,076	54.89	94.63
GS007-21b	Gansu, China	Leaf	22,900,431	6,846,977,174	55.11	94.63
GS007-21c	Gansu, China	Leaf	21,390,448	6,387,744,030	54.47	94.61
NM037-CKa	Inner Mongolia, China	Leaf	25,719,380	7,670,389,794	52.78	94.96
NM037-CKb	Inner Mongolia, China	Leaf	20,463,253	6,116,544,846	55.5	94.93
NM037-CKc	Inner Mongolia, China	Leaf	23,053,582	6,890,241,856	55.74	94.85
NM037-3a	Inner Mongolia, China	Leaf	22,779,870	6,799,450,890	55.32	94.64
NM037-3b	Inner Mongolia, China	Leaf	22,855,065	6,826,781,922	55.22	94.97
NM037-3c	Inner Mongolia, China	Leaf	20,511,306	6,123,180,168	54.19	94.45
NM037-9a	Inner Mongolia, China	Leaf	23,578,021	7,044,303,148	55.07	94.59
NM037-9b	Inner Mongolia, China	Leaf	21,833,233	6,521,644,198	54.25	94.45
NM037-9c	Inner Mongolia, China	Leaf	22,846,423	6,823,134,584	53.27	94.8
NM037-21a	Inner Mongolia, China	Leaf	22,418,274	6,689,697,678	53.36	94.31
NM037-21b	Inner Mongolia, China	Leaf	20,602,377	6,158,168,132	52.96	94.8
NM037-21c	Inner Mongolia, China	Leaf	22,036,643	6,569,000,196	48.17	94.54
QH009-CKa	Qinghai, China	Leaf	22,810,838	6,815,889,282	55.33	94.71
QH009-CKb	Qinghai, China	Leaf	26,159,874	7,813,380,624	54.95	94.33
QH009-CKc	Qinghai, China	Leaf	30,127,905	8,999,774,630	54.88	95
QH009-3a	Qinghai, China	Leaf	31,553,844	9,419,343,524	55.29	94.92
QH009-3b	Qinghai, China	Leaf	23,023,445	6,879,021,946	55.53	94.81
QH009-3c	Qinghai, China	Leaf	29,479,626	8,810,310,292	54.28	94.63
QH009-9a	Qinghai, China	Leaf	22,268,617	6,658,769,864	55.77	94.63

QH009-9b	Qinghai, China	Leaf	21,036,278	6,286,880,194	55.43	94.42
QH009-9c	Qinghai, China	Leaf	22,000,234	6,568,525,796	55.53	94.58
QH009-21a	Qinghai, China	Leaf	24,738,482	7,388,283,510	54.16	94.62
QH009-21b	Qinghai, China	Leaf	24,530,546	7,326,814,576	54.65	94.42
QH009-21c	Qinghai, China	Leaf	30,558,461	9,126,116,402	54.63	94.87

Clean reads: total read number of clean sequencing data; Clean bases: total bases of clean sequencing data; GC(%): GC content; Q30(%): percentage of bases with Phred quality score ≥ 30. The UV-B irradiation treatment was applied to GS007, NM037, and QH009 for 21 days, with leaf samples collected on days 3, 9, and 21 of treatment for RNA sequencing. Lowercase letters a, b, and c represent three biological replicates.

Supplementary Table 10 RNA-seq data following drought treatment

Sample ID	Geography origin	Tissue	Clean reads	Clean bases	GC (%)	Q30 (%)
GS005-CKa	Gansu, China	Leaf	19,673,841	5,883,327,992	54.87	95.4
GS005-CKb	Gansu, China	Leaf	19,434,668	5,813,156,662	55.95	95.16
GS005-CKc	Gansu, China	Leaf	19,304,357	5,774,027,658	54.63	95.55
GS005-7a	Gansu, China	Leaf	19,650,531	5,874,333,480	55.09	95.42
GS005-7b	Gansu, China	Leaf	20,896,176	6,253,424,320	54.8	94.77
GS005-7c	Gansu, China	Leaf	21,904,376	6,549,613,724	54.51	95.17
GS005-14a	Gansu, China	Leaf	21,216,066	6,344,793,650	55.58	95.08
GS005-14b	Gansu, China	Leaf	21,806,173	6,517,372,686	55.82	95.61
GS005-14c	Gansu, China	Leaf	21,246,582	6,352,761,906	55.48	94.98
GS005-28a	Gansu, China	Leaf	19,723,542	5,899,559,162	55.25	95
GS005-28b	Gansu, China	Leaf	20,956,655	6,265,646,484	54.64	93.96
GS005-28c	Gansu, China	Leaf	19,574,928	5,853,992,362	54.66	95.35
NM037-CKa	Inner Mongolia, China	Leaf	21,461,419	6,420,143,906	56.07	95.11
NM037-CKb	Inner Mongolia, China	Leaf	20,654,769	6,161,275,338	55.83	95.29
NM037-CKc	Inner Mongolia, China	Leaf	22,964,293	6,850,319,234	55.52	95.7
NM037-7a	Inner Mongolia, China	Leaf	23,319,701	6,971,496,024	53.94	95.5
NM037-7b	Inner Mongolia, China	Leaf	22,168,047	6,624,245,332	54.7	95.78
NM037-7c	Inner Mongolia, China	Leaf	23,185,213	6,923,809,724	53.31	94.88
NM037-14a	Inner Mongolia, China	Leaf	19,315,572	5,771,452,406	55.39	95.36
NM037-14b	Inner Mongolia, China	Leaf	20,155,075	6,030,102,440	57.08	95.26
NM037-14c	Inner Mongolia, China	Leaf	21,399,993	6,401,366,490	55.17	94.96
NM037-28a	Inner Mongolia, China	Leaf	19,652,005	5,874,870,112	54.55	95.65
NM037-28b	Inner Mongolia, China	Leaf	19,425,117	5,808,850,424	55.04	95.23
NM037-28c	Inner Mongolia, China	Leaf	23,565,007	7,046,070,740	55.59	95.51
SC020-CKa	Sichuan, China	Leaf	22,098,031	6,607,718,054	53.1	95.79
SC020-CKb	Sichuan, China	Leaf	25,204,533	7,532,576,856	54.09	95.41
SC020-CKc	Sichuan, China	Leaf	22,703,681	6,787,516,058	54.3	95.74
SC020-7a	Sichuan, China	Leaf	19,777,703	5,910,832,864	54.26	95.44
SC020-7b	Sichuan, China	Leaf	20,965,718	6,264,941,130	54.16	95.07

SC020-7c	Sichuan, China	Leaf	22,006,140	6,581,518,566	54.04	95.44
SC020-14a	Sichuan, China	Leaf	20,950,384	6,261,683,148	54.64	95.42
SC020-14b	Sichuan, China	Leaf	20,312,296	6,029,006,620	50.62	95.59
SC020-14c	Sichuan, China	Leaf	19,628,297	5,867,276,338	55.64	95.56
SC020-28a	Sichuan, China	Leaf	21,246,801	6,353,525,170	55.45	95.38
SC020-28b	Sichuan, China	Leaf	26,207,972	7,846,372,174	55	95.25
SC020-28c	Sichuan, China	Leaf	19,249,463	5,753,325,146	55.51	95.27

Clean reads: total read number of clean sequencing data; Clean bases: total bases of clean sequencing data; GC(%): GC content; Q30(%): percentage of bases with Phred quality score ≥ 30. The drought treatment was applied to GS005, NM037, and SC020 for 28 days, with leaf samples collected on days 7, 14, and 28 of treatment for RNA sequencing. Lowercase letters a, b, and c represent three biological replicates.

Supplementary Table 11 Correlation between RNA-seq transcript abundance and qRT-PCR relative expression levels under UV-B and drought treatments

Treatment	Gene id	Primer sequences 5'-3'	r^2	Treatment	Gene id	Primer sequences 5'-3'	r^2
UV-B	EVM0001149	F: AATGAGAAGATGGAGCTCATGC<br>R: GTGATCTTACATCATCACTCTCTTCC	0.87	Drought	EVM0068112	F: TACCAGGCCTTTGAGCTCATC<br>R: CTTGAACCAAACGGCCTCG	0.88
	EVM0021772	F: AGAAGGACCCGGAGAAGAA<br>R: GTACCGTTCTTGATCTCCTTGAG	0.93		EVM0135475	F: ATGGTTGCTATGCTTGGC<br>R: CAAGACAAGGTTAGTGCATGAATG	0.84
	EVM0000864	F: CTTGGTGAGATCGTCGACC<br>R: AGAACCCGAACATGGAGAAC	0.94		EVM0074266	F: GCCATTGCAACAAAGTTTGC<br>R: TGGAGTTGGTATGACTCAACC	0.95
	EVM0002439	F: CTCCGACCATTGGCATTTCT<br>R: TACCTTGACCTTGCAAGCAC	0.69		EVM0088380	F: AGGAGAGGAGAGCGATGG<br>R: ATGCCGAGCTTGCTCAC	1.00
	EVM0003293	F: TTTCCAACACTCCTGCAC<br>R: CGTTCTCACCTGAACATCC	0.78		EVM0092067	F: CTCTTCATCGTGGAGCTCATC<br>R: ACTGTGTGTACCCGACGTC	0.84
	EVM0020232	F: TGCATTGTCAGCCAAGATCTG<br>R: TTGATCTTGGAAGCCCTCTTG	0.78		EVM0129929	F: CAGAGGTTCCGCGAGTG<br>R: TACATGCATGAGATCGAGACG	0.48
	EVM0050760	F: CACTCTGGTTAGTCACCAGGA<br>R: TCTCGTCCATCGACAAGTTG	0.68		EVM0031560	F: GTGGAGACCTGGAGGTTATAGA<br>R: GCACAGGGTTACGAAGTTGA	0.92
	EVM0092577	F: GCCACCAACAGGATCATCA<br>R: GCTGCTTGATCTCAGCCTT	0.73		EVM0001544	F: GGTCGATTGAGGGATTCAGTAC<br>R: GCATTGGCATATGTTGCGATC	0.61
	Mean		0.79		Mean		0.81

Supplementary Table 12 Distribution of GST subfamily members across different species and subgenomes

Species/Subgenome	Number of GST subfamily members									
	phi	tau	lambda	theta	zeta	DHAR	TCHQD	METAXIN	EF1Bγ	Total
A. thaliana	23	31	5	5	5	4	3	2	4	82
E. nutans-H	31	79	0	1	4	2	1	1	0	119
E. nutans-St	31	88	1	5	5	2	1	1	0	134
E. nutans-Y	30	49	0	1	4	2	2	1	0	89
E. sibiricus-H	29	49	1	1	4	2	3	2	0	91
E. sibiricus-St	25	67	4	3	5	1	0	2	0	107
H. vulgare-H	29	64	4	1	3	2	2	1	0	106
H. vulgare var. *nudum*-H	20	56	2	1	3	2	1	2	0	87
T. aestivum-A	27	68	0	3	4	2	1	1	0	106
T. aestivum-B	34	67	0	6	5	2	1	1	0	116
T. aestivum-D	33	69	0	3	4	1	1	1	0	112

Supplemental materials and methods

Genome sequencing

Fresh leaves were collected from an individual plant (*Elymus nutans* 'Aba') at the 3-leaf stage at Sichuan Academy of Grassland Sciences, Hongyuan County, Sichuan Province, China (102.54°E, 32.79°N, 3495.86 m) and used for genome sequencing. Briefly, genomic DNA was extracted from the samples using a modified CTAB method (Agbagwa *et al.*, 2012) and quantified using a Qubit 2.0 Fluorometer (Life Technologies, CA, USA) and an Agilent 2100 Bioanalyzer (Agilent Technologies, CA, USA). To generate short reads, two libraries were separately constructed with insert sizes of 270 bp and 8 kb using a TruSeq Nano DNA Library Prep Kit (Illumina, CA, USA) and an SQK-LSK108 Sequencing kit [Oxford Nanopore Technology (ONT), UK], respectively, and sequenced on an Illumina NovaSeq 6000 instrument. To generate long reads, the BluePippin™ System was first used to obtain large DNA segments of >30 kb, and a large-segment DNA library was prepared using an ONT Template prep kit (SQK-LSK109) and a NEB Next FFPE DNA Repair Mix kit (New England Biolabs, MA, USA). The high-quality library was sequenced on the ONT PromethION platform with Corresponding R9 cell and ONT Sequencing Reagents kit (EXP-FLP001.PRO.6). Hi-C libraries with insert sizes of 300–700 bp were constructed following a standard protocol described previously and sequenced on an Illumina HiSeq 4000 instrument.

Fresh tissues (leaf, stem, flower, seed, and root tissue) were collected from the same plant for total RNA extraction using a Plant total RNA Kit (TIANGEN, China). RNA-seq libraries were constructed using a TruSeq RNA Sample Preparation kit (Illumina) and sequenced on an Illumina HiSeq 4000 instrument. Raw sequencing data were processed by trimming adaptors and filtering out low-quality reads to generate clean data, which were used for annotation of protein-coding genes.

Genome survey and assembly

A genome survey of *E. nutans* was performed using methods based on flow cytometry and *k*-mer frequency. *E. nutans* seeds were germinated in an incubator (germination conditions were set as follows: 25/20°C (day/night) under a 12-h light/12-h dark cycle, a light intensity of 225 ± 25 μmol

m^{-2} s^{-1} and a relative humidity of 70% ± 5%), and root tips were excised from the seedlings and treated using the conventional pressing method (Yang *et al.*, 2017) for karyotype analysis and chromosome counting. Genome size and ploidy were analyzed using a CyFlow Cube6 Flow Cytometer (Sysmex Partec, Germany) using *Triticum aestivum* 'Chinese Spring' as a reference (Dolezel and Bartos, 2005). The Illumina short reads were used to estimate genome size, heterozygosity, and repeat content through *k*-mer frequency analysis (k = 23) using Jellyfish (Liu *et al.*, 2013). The inferred size of the *E. nutans* genome is 10.50 Gb, with 0.01% heterozygosity and 78.00% repeat sequences.

De novo assembly of the *E. nutans* genome was based on ONT long reads. The ONT long reads were first corrected using Canu v1.7.1 with the parameter ErrorRate=0.025 (Koren *et al.*, 2017), and the initial genome assembly was constructed using WTDBG v2.5 (Ruan and Li, 2020). The initial assembly was corrected three times using Recon (Vaser *et al.*, 2017) based on the ONT long reads. The Illumina short reads were mapped to the initial assembly using the "MEM" module of BWA v0.7.10 (Li and Durbin, 2009), and three rounds of polishing were performed using Pilon with the parameters --mindepth 10 --changes --threads 4 --fix bases (Walker *et al.*, 2014).

To construct pseudochromosomes, Hi-C raw data were trimmed using LACHESIS v2.0 (Burton *et al.*, 2013). The high-quality Hi-C reads were then aligned to the draft assembly using BWA software, and uniquely mapped reads were selected by HiC-Pro v.2.10.0 (Servant *et al.*, 2015) for further analysis. The manually corrected scaffolds were placed into 21 pseudochromosomes using LACHESIS software with the following parameters: CLUSTER_MIN_RE_SITES=283, CLUSTER_MAX_LINK_DENSITY=2, ORDER_MIN_N_RES_IN_TRUNK=192, ORDER_MIN_N_RES_IN_SHREDS=178. Placement and orientation errors exhibiting obvious discrete chromatin interaction patterns were manually adjusted. The Hi-C read mapping and the quality of the final chromosome-level assembly were assessed using HiC-Pro software.

The completeness, of the final genome assembly was assessed using BUSCO v.5.0 (Simão *et al.*, 2015) against the Embryophyta dataset with default parameters. Alignment and coverage of ONT long clean reads and Illumina short clean reads against the final genome assembly were evaluated

using BWA software. LTR assembly index (LAI) was used to evaluate the continuity of the assembly based on full-length long terminal repeat retrotransposons (LTR-RTs) (Ou *et al.*, 2018).

Centromere and sub-genome identification

A reference-guided strategy based on the sequence collinearity of the subgenomes of *Hordeum vulgare* (HH) and *Elymus sibiricus* (StStHH) was used to distinguish subgenomes H, St, and Y of *E. nutans*. The 21 chromosomes were mapped to the *H. vulgare* genome using MUMmer v.3.0 (Kurtz *et al.*, 2004) and clustered into seven groups, each containing three chromosomes. The seven chromosomes with the best alignments were selected as those from the H subgenome. Similarly, the 14 remaining chromosomes of *E. nutans* from subgenomes St and Y were mapped onto the H and St subgenomes of *E. sibiricus* (Yan *et al.*, 2024) to identify the seven chromosomes from St, with the seven remaining chromosomes presumed to be from the Y subgenome. Overall, the *E. nutans* genome was resolved for the chromosomes and their numbers from subgenomes St, Y, and H.

The Cereba/Quinta sequences in *Triticum aestivum* were downloaded from NCBI (GenBank accession no. FN564437.1) to identify long terminal repeats in the *E. nutans* genome using RepeatMaster v.4.1.5 (http://www.repeatmasker.org/). The maize T2T genome assembly was downloaded (Chen *et al.*, 2023), and the sequences in the centromere regions were retrieved based on published information. The maize centromeric sequences were aligned to the *E. nutans* genome, and the sequence hits were identified and summarized using a bin size of 1 Mb. The density distributions of the sequence hits were plotted onto the chromosomes, with the peaks used to determine the positions of the centromeres. The centromeric regions identified by two methods were combined to extract sequences, which were then analyzed using the Tandem Repeats Finder (TRF) software. Tandem repeats were classified into three types based on the consensus size: microsatellites (ConsensusSize $<$ 10), minisatellites (10 $\leq$ ConsensusSize $<$ 100), and satellites (ConsensusSize $\geq$ 100). The centromeric sequences of each subgenome were analyzed, and the proportion of each repeat type was calculated.

Annotation of repetitive sequences and protein-coding genes

Repetitive sequences including tandem repeats and transposable elements (TEs) were identified

from the whole genome of *E. nutans*. Tandem repeats were identified using GMATA v2.2 (Wang and Wang, 2016) and Tandem Repeats Finder v4.07b (Benson, 1999). A species-specific *de novo* repeat library was constructed using MITE-Hunter (Han and Wessler, 2010), LTR_FINDER v1.0.5 (Xu and Wang, 2007), and RepeatModeler v2.0.1 (https://github.com/Dfam-consortium/RepeatModeler). RepeatMasker software was then used to search for TEs against Repbase v19.06 (Jurka, 2005) and the species-specific *de novo* repeat library.

Protein-coding genes were predicted using an evidence-based annotation workflow by integrating evidence from *de novo* prediction, homology searches, and transcriptomic data. The *de novo* gene models were predicted using Augustus v.2.4 (Stanke *et al.*, 2008) and SNAP software (Korf, 2004). For the homology-based approach, GeMoMa v.1.7 (Keilwagen *et al.*, 2018) software was employed using reference gene models from other species, including *Arabidopsis thaliana*, *H. vulgare*, *Oryza sativa*, *Sorghum bicolor*, *T. aestivum*, and *Zea mays*. For transcript-based prediction, clean RNA-seq data were mapped to the *E. nutans* genome using HISAT v.2.0.4 (Kim *et al.*, 2015) and assembled using StringTie v.1.2.3 (Pertea *et al.*, 2015). GeneMarkS-T v.5.1 (Tang *et al.*, 2015) was used to predict genes based on the assembled transcripts. PASA v.2.0.2 (Haas *et al.*, 2008) was used to predict genes based on the unigenes assembled by Trinity v.2.11 (Grabherr *et al.*, 2011). Gene models from these different approaches were combined using EVidenceModeler v.1.1.1 (Haas *et al.*, 2008) and updated using PASA software after removing TE-related genes, pseudogenes, and noncoding genes using TransposonPSI v1.0 (Urasaki *et al.*, 2017) with default settings.

The final gene models were annotated by searching the GenBank Non-Redundant (NR, data: 20200921), TrEMBL (data: 202005), Pfam v.33.1, SwissProt (data: 202005), eukaryotic orthologous groups (KOG, data: 20110125), and Kyoto Encyclopedia of Genes and Genomes (KEGG, data: 20191220) databases using BLAST software. Gene ontology (GO, data: 20200615) categories were annotated using Blast2go v.5.2.5 (Conesa *et al.*, 2005) based on the NR annotation results.

Comparative genomic analysis

The protein sequences of *E. nutans* (StStYYHH), *E. sibiricus* (StStHH, NGDC BioProject

accession number: PRJCA029280), *H. vulgare* (HH)(Beier *et al.*, 2017), *Aegilops tauschii* (DD)(Luo *et al.*, 2017), *T. aestivum* B subgenome (BB)(https://urgi.versailles.inra.fr/download/iwgsc/IWGSC_RefSeq_Annotations/v1.1/), *Dasypyrum villosum* (VV)(Zhang *et al.*, 2023), *Thinopyrum elongatum* (EE) (Wang *et al.*, 2020), *Thinopyrum intermedium* (https://phytozome-next.jgi.doe.gov/info/Tintermedium_v3_1), *Secale cereale* (RR)(Li *et al.*, 2021), *Triticum urartu* (AA)(Ling *et al.*, 2018), *H. vulgare* var. *nudum* (HH)(https://ftp.ncbi.nlm.nih.gov/genomes/all/GCA/004/114/815/GCA_004114815.1_Hulless_Barley_ass.V2/), *Brachypodium distachyon* (http://plants.ensembl.org/Brachypodium_distachyon/Info/Index), and *O. sativa* (as the outgroup, https://rice.uga.edu/pub/data/Eukaryotic_Projects/o_sativa/) were aligned using Diamond v.0.9.29 (Buchfink *et al.*, 2021) with an E-value of 0.001 using the all-vs-all strategy. The orthologous and paralogous gene families were identified by OrthoFinder v.2.4.0 (Emms and Kelly, 2019) and annotated using PANTHER v.14 (Mi *et al.*, 2019). The protein sequences of single-copy orthologs were aligned using MAFFT v.7.205 (Nakamura *et al.*, 2018), and a phylogenetic tree was reconstructed using IQ-TREE v.1.6.11 (Minh *et al.*, 2020) based on the best evolution model JTT+F+I+G4 identified by ModelFinder (Kalyaanamoorthy *et al.*, 2017) with 1000 bootstrap replicates. The divergence times were estimated using the MCMCtree program of PAML v.4.9 (Yang, 2007) with the parameters: burnin = 5000000, sampfreq=30, nsample=10000000. Expansion and contraction analysis of the gene families was performed using CAFE v4.2102 (Han *et al.*, 2013). Three calibration points (*A. tauschii* vs. *O. sativa*: 42–52 MYA, *B. distachyon* vs. *A. tauschii*: 26–39 MYA, *T. aestivum* B subgenome vs. *T. urartu*: 2.6–5.3 MYA) were derived from the TimeTree database (http://www.timetree.org/).

Syntenic relationship among the subgenomes of *E. nutans* and the related species *H. vulgare*, *H. marinum, T. intermedium*, *D. villosum, P. libanotica*, *E. sibiricus*, *T. elongatum*, *S. cereale*, *T. aestivum*, *T. urartu*, and *A. tauschii* were investigated using jcvi software (https://github.com/tanghaibao/jcvi/wiki). The Ks distribution curve for species-specific and interspecies homologous blocks was constructed using the WGDI software v. 0.6.5 (Sun *et al.*, 2022). The *Ks* values corresponding to the common ancient whole genome duplication (WGD) event in grasses were estimated to range from 0.70 to 0.82, based on the interspecies homologous blocks. A

speciation peak was observed at *Ks* = 0.52 for *Aegilops tauschii* and *Oryza*. To estimate the timing of the common WGD event for grasses, we utilized the divergence time of approximately 46.51 million years ago between *A. tauschii* and *O. sativa* (from www.timetree.org). This divergence time allowed us to infer the WGD event to have occurred between 62.61 million years ago and 73.34 million years ago.

Sequence similarities of reads from different *Elymus* species, including *Elymus alaskanus* (PRJEB43865), *Elymus alaskanus* subsp. *Borealis* (PRJEB43865), *Elymus alaskanus* subsp. *Hyperarcticus* (PRJEB43865), *Elymus caninus* (PRJEB43865), *Elymus caninus* var. *muticus* (PRJEB43865), *Elymus fibrosus* (PRJEB43865), *Elymus macrourus* (PRJEB43865), *Elymus mutabilis* (PRJEB43865), *Elymus sibiricus* (PRJEB43865), *Elymus trachycaulus* subsp. *Virescens* (PRJEB43865), *Elymus violaceus* (PRJEB43865), *Elymus repens* (PRJNA607895), *Elymus magellanicus* (PRJNA783482), *Pseudorogneria libanotica* (PRJNA843189), *Pseudorogneria stipifolia* (PRJNA843189), *Pseudorogneria tauri* (PRJNA843189), *Pseudoroegneria spicata* (PRJNA843189), and *Pseudoroegneria strigose* (PRJNA843189) that were uniquely mapped to the H, St and Y subgenomes of *E. nutans*. For each accession, we randomly selected clean paired-end reads with 1× coverage from resequencing data. These reads were mapped to the repeat-masked 'Aba' reference genome using BWA with default parameters. Uniquely mapped reads were filtered with SAMtools (v1.9). The best hit for each read was selected if the BLASTn score was at least 15 higher than the next best hit, with query coverage >60 bp. The average identity over a 20 Mb sliding window was calculated and plotted along the *E. nutans* 'Aba' chromosomes with a 1 Mb step size.

UV treatment, drought treatment, and RNA-seq

Based on preliminary evaluation and screening, five *E. nutans* accessions (QH009, GS007, NM037, GS005, and SC020; Supplementary Table 6) were grown in a climate-controlled chamber at the optimal temperature of 25/20°C (day/night) under a 12-h light/12-h dark cycle, a light intensity of 225 ± 25 μmol m^{-2} s^{-1} and a relative humidity of 70% ± 5% (Han *et al.*, 2024). UV-B radiation treatment and drought stress treatment were performed at 30 days after seed germination: QH009, GS007, and NM037 were treated with UV-radiation; NM037, GS005, and SC020 were treated with drought. Seedlings were exposed to UV-B radiation between 10:30 and 14:30 daily at an intensity

of approximately 200 μW/cm^2 (105% of the UV intensity in the Tibetan Plateau). UV-B irradiation treatment was performed for 21 days, and leaf samples were collected on days 3, 9, and 21 of irradiation treatment; untreated samples served as a control. In the drought stress experiment, drought treatment was performed for 28 days, and leaf samples were collected on days 7, 14, and 28 of treatment. Samples from plants at the same stage of growth without drought treatment were used as a control. Each treatment was performed with three plants. All samples were quickly frozen in liquid nitrogen for transcriptome sequencing. RNA-seq libraries were constructed using a TruSeq RNA Sample Preparation kit (Illumina) and sequenced on an Illumina HiSeq 4000 instrument.

Transcriptomic analysis of genes related to drought and UV tolerance

The clean RNA-seq data were mapped to the *E. nutans* genome using HISAT software, and fragments per kilobase of transcript per million fragments mapped (FPKM) values were calculated to estimate expression abundance using StringTie software. Differentially expressed genes (DEGs) were identified using DESeq2 v1.6.3 (Love *et al.*, 2014) with a false discovery rate (FDR) < 0.05 and fold change (FC) ≥ 5. DEGs significantly associated with drought and UV tolerance were selected as candidate genes using WGCNA v1.47 (Langfelder and Horvath, 2008).

To validate our RNA-seq results, we conducted qRT-PCR experiments on eight randomly selected genes. Total RNA was reverse transcribed into cDNA using the RevertAid First Strand cDNA Synthesis Kit (Vazyme, China). Real-time quantitative PCR was performed with the ChamQ Universal SYBR qPCR Master Mix (Vazyme, China) on a Light Cycler 480 RT-PCR System (Roche Applied Science, Germany). Gene expression levels were calculated using the $2^{-\Delta\Delta CT}$ method (Livak and Schmittgen, 2001), with three biological replicates for each treatment.

To predict the transcription factor (TF) binding sites in the promoters of these candidate genes, 2-kb sequences upstream of the genes were extracted from the PlantTFDB website based on *A. thaliana* promoter sequences. The TF binding sites were predicted using JASPAR v.2018 (Khan *et al.*, 2018) and TFBSTools (https://github.com/ge11232002/TFBSTools) against the *A. thaliana* promoter sequences. The correlation of TFs and candidate genes from among the DEGs related to drought and UV tolerance was calculated using Hmisc v.5.1 (https://cran.r-

project.org/web/packages/Hmisc/index.html). The co-expression regulatory network between the TFs and candidate gene pairs ($r > 0.8$, $p < 0.05$) was visualized using Cytoscape v3.6.1 (Otasek *et al.*, 2019).

Analysis of GST gene family members related to drought and UV tolerance

The sequences of glutathione S-transferase (GST) gene family members from *A. thaliana* were downloaded from The Arabidopsis Information Resource (TAIR, https://www.arabidopsis.org/). All *GST* genes in the *E. nutans* genome were searched against the *A. thaliana* sequences using BLAST software, and the domains of the candidate genes were identified by HMMER v3.2.1 (Finn *et al.*, 2011) by searching the Pfam database (Punta *et al.*, 2012).

REFERENCES

Agbagwa, I.O., Datta, S., Patil, P.G., Singh, P., and Nadarajan, N. (2012) A protocol for high-quality genomic DNA extraction from legumes. *Genet. Mol. Res. GMR*, **11**, 4632–4639.

Beier, S., Himmelbach, A., Colmsee, C., Zhang, X.-Q., Barrero, R.A., Zhang, Q., et al. (2017) Construction of a map-based reference genome sequence for barley, *Hordeum vulgare* L. *Sci. Data*, **4**, 170044.

Benson, G. (1999) Tandem repeats finder: a program to analyze DNA sequences. *Nucleic Acids Res.*, **27**, 573–580.

Buchfink, B., Reuter, K., and Drost, H. G. (2021) Sensitive protein alignments at tree-of-life scale using DIAMOND. *Nat. Methods*, **18**, 366–368.

Burton, J.N., Adey, A., Patwardhan, R.P., Qiu, R., Kitzman, J.O., and Shendure, J. (2013) Chromosome-scale scaffolding of de novo genome assemblies based on chromatin interactions. *Nat. Biotechnol.*, **31**, 1119–1125.

Chen, J., Wang, Z., Tan, K., Huang, W., Shi, J., Li, T., et al. (2023) A complete telomere-to-telomere assembly of the maize genome. *Nat. Genet.*, **55**, 1221–1231.

Conesa, A., Götz, S., García-Gómez, J.M., Terol, J., Talón, M., and Robles, M. (2005) Blast2GO: a universal tool for annotation, visualization and analysis in functional genomics research. *Bioinforma. Oxf. Engl.*, **21**, 3674–3676.

Dolezel, J. and Bartos, J. (2005) Plant DNA flow cytometry and estimation of nuclear genome size. *Ann. Bot.*, **95**, 99–110.

Emms, D.M. and Kelly, S. (2019) OrthoFinder: phylogenetic orthology inference for comparative genomics. *Genome Biol.*, **20**, 238.

Finn, R.D., Clements, J., and Eddy, S.R. (2011) HMMER web server: interactive sequence similarity searching. *Nucleic Acids Res.*, **39**, W29-37.

Grabherr, M.G., Haas, B.J., Yassour, M., Levin, J.Z., Thompson, D.A., Amit, I., et al. (2011) Trinity: reconstructing a full-length transcriptome without a genome from RNA-Seq data. *Nat. Biotechnol.*, **29**, 644–652.

Haas, B.J., Salzberg, S.L., Zhu, W., Pertea, M., Allen, J.E., Orvis, J., et al. (2008) Automated eukaryotic gene structure annotation using EVidenceModeler and the Program to Assemble Spliced Alignments. *Genome Biol.*, **9**, R7.

Han, M., Chen, Z., Sun, G., Feng, Y., Guo, Y., Bai, S., and Yan, X. (2024) Nano-Fe3O4: Enhancing the tolerance of *Elymus nutans* to Cd stress through regulating programmed cell death. *Environ. Pollut.*, **360**, 124711.

Han, M.V., Thomas, G.W.C., Lugo-Martinez, J., and Hahn, M.W. (2013) Estimating gene gain and loss rates in the presence of error in genome assembly and annotation using CAFE 3. *Mol. Biol. Evol.*, **30**, 1987–1997.

Han, Y. and Wessler, S.R. (2010) MITE-Hunter: a program for discovering miniature inverted-repeat transposable elements from genomic sequences. *Nucleic Acids Res.*, **38**, e199.

Kalyaanamoorthy, S., Minh, B.Q., Wong, T.K.F., von Haeseler, A., and Jermiin, L.S. (2017) ModelFinder: fast model selection for accurate phylogenetic estimates. *Nat. Methods*, **14**, 587–589.

Keilwagen, J., Hartung, F., Paulini, M., Twardziok, S.O., and Grau, J. (2018) Combining RNA-seq data and homology-based gene prediction for plants, animals and fungi. *BMC Bioinformatics*, **19**, 189.

Khan, A., Fornes, O., Stigliani, A., Gheorghe, M., Castro-Mondragon, J.A., van der Lee, R., et al. (2018) JASPAR 2018: update of the open-access database of transcription factor binding profiles and its web framework. *Nucleic Acids Res.*, **46**,

D260–D266.

Kim, D., Langmead, B., and Salzberg, S.L. (2015) HISAT: a fast spliced aligner with low memory requirements. *Nat. Methods*, **12**, 357–360.

Koren, S., Walenz, B.P., Berlin, K., Miller, J.R., Bergman, N.H., and Phillippy, A.M. (2017) Canu: scalable and accurate long-read assembly via adaptive k-mer weighting and repeat separation. *Genome Res.*, **27**, 722–736.

Korf, I. (2004) *Gene finding in novel genomes*. *BMC Bioinformatics*, **5**, 59.

Kurtz, S., Phillippy, A., Delcher, A.L., Smoot, M., Shumway, M., Antonescu, C., and Salzberg, S.L. (2004) Versatile and open software for comparing large genomes. *Genome Biol.*, **5**, R12.

Langfelder, P. and Horvath, S. (2008) WGCNA: an R package for weighted correlation network analysis. *BMC Bioinformatics*, **9**, 559.

Li, G., Wang, L., Yang, J., He, H., Jin, H., Li, X., et al. (2021) A high-quality genome assembly highlights rye genomic characteristics and agronomically important genes. *Nat. Genet.*, **53**, 574–584.

Li, H. and Durbin, R. (2009) Fast and accurate short read alignment with Burrows-Wheeler transform. *Bioinforma. Oxf. Engl.*, **25**, 1754–1760.

Ling, H.-Q., Ma, B., Shi, X., Liu, H., Dong, L., Sun, H., et al. (2018) Genome sequence of the progenitor of wheat A subgenome *Triticum urartu*. *Nature*, **557**, 424–428.

Liu, B., Shi, Y., Yuan, J., Hu, X., Zhang, H., Li, N., et al. (2013) Estimation of genomic characteristics by analyzing k-mer frequency in de novo genome projects. *ArXiv Genomics*.

Livak, K.J. and Schmittgen, T.D. (2001) Analysis of Relative Gene Expression Data Using Real-Time Quantitative PCR and the 2−ΔΔCT Method. *Methods*, **25**, 402–408.

Love, M.I., Huber, W., and Anders, S. (2014) Moderated estimation of fold change and dispersion for RNA-seq data with DESeq2. *Genome Biol.*, **15**, 550.

Luo, M.-C., Gu, Y.Q., Puiu, D., Wang, H., Twardziok, S.O., Deal, K.R., et al. (2017) Genome sequence of the progenitor of the wheat D genome *Aegilops tauschii*. *Nature*, **551**, 498–502.

Mi, H., Muruganujan, A., Ebert, D., Huang, X., and Thomas, P.D. (2019) PANTHER version 14: more genomes, a new PANTHER GO-slim and improvements in enrichment analysis tools. *Nucleic Acids Res.*, **47**, D419–D426.

Minh, B.Q., Schmidt, H.A., Chernomor, O., Schrempf, D., Woodhams, M.D., von Haeseler, A., and Lanfear, R. (2020) IQ-TREE 2: New Models and Efficient Methods for Phylogenetic Inference in the Genomic Era. *Mol. Biol. Evol.*, **37**, 1530–1534.

Nakamura, T., Yamada, K.D., Tomii, K., and Katoh, K. (2018) Parallelization of MAFFT for large-scale multiple sequence alignments. *Bioinforma. Oxf. Engl.*, **34**, 2490–2492.

Otasek, D., Morris, J.H., Bouças, J., Pico, A.R., and Demchak, B. (2019) Cytoscape Automation: empowering workflow-based network analysis. *Genome Biol.*, **20**, 185.

Ou, S., Chen, J., and Jiang, N. (2018) Assessing genome assembly quality using the LTR Assembly Index (LAI). *Nucleic Acids Res.*, **46**, e126.

Pertea, M., Pertea, G.M., Antonescu, C.M., Chang, T. C., Mendell, J.T., and Salzberg, S.L. (2015) StringTie enables improved reconstruction of a transcriptome from RNA-seq reads. *Nat. Biotechnol.*, **33**, 290–295.

Punta, M., Coggill, P.C., Eberhardt, R.Y., Mistry, J., Tate, J., Boursnell, C., et al. (2012) The Pfam protein families database. *Nucleic Acids Res.*, **40**, D290-301.

Ruan, J. and Li, H. (2020) Fast and accurate long-read assembly with wtdbg2. *Nat. Methods*, **17**, 155–158.

Servant, N., Varoquaux, N., Lajoie, B. R., Viara, E., Chen, C.-J., Vert, J. P., Heard, E., Dekker, J., and Barillot, E. (2015). HiC-Pro: an optimized and flexible pipeline for Hi-C data processing. *Genome Biol.* **16**:259.

Simão, F.A., Waterhouse, R.M., Ioannidis, P., Kriventseva, E.V., and Zdobnov, E.M. (2015) BUSCO: assessing genome assembly and annotation completeness with single-copy orthologs. *Bioinforma. Oxf. Engl.*, **31**, 3210–3212.

Stanke, M., Diekhans, M., Baertsch, R., and Haussler, D. (2008) Using native and syntenically mapped cDNA alignments to improve de novo gene finding. *Bioinforma. Oxf. Engl.*, **24**, 637–644.

Sun, P., Jiao, B., Yang, Y., Shan, L., Li, T., Li, X., et al. (2022) WGDI: A user-friendly

toolkit for evolutionary analyses of whole-genome duplications and ancestral karyotypes. *Mol. Plant*, **15**, 1841–1851.

Tang, S., Lomsadze, A., and Borodovsky, M. (2015) Identification of protein coding regions in RNA transcripts. *Nucleic Acids Res.*, **43**, e78.

Urasaki, N., Takagi, H., Natsume, S., Uemura, A., Taniai, N., Miyagi, N., et al. (2017) Draft genome sequence of bitter gourd (Momordica charantia), a vegetable and medicinal plant in tropical and subtropical regions. DNA Res. Int. J. Rapid Publ. Rep. *Genes Genomes*, **24**, 51–58.

Vaser, R., Sović, I., Nagarajan, N., and Šikić, M. (2017) Fast and accurate de novo genome assembly from long uncorrected reads. *Genome Res.*, **27**, 737–746.

Walker, B.J., Abeel, T., Shea, T., Priest, M., Abouelliel, A., Sakthikumar, S., et al. (2014) Pilon: an integrated tool for comprehensive microbial variant detection and genome assembly improvement. *PloS One*, **9**, e112963.

Wang, H., Sun, S., Ge, W., Zhao, L., Hou, B., Wang, K., et al. (2020) Horizontal gene transfer of Fhb7 from fungus underlies Fusarium head blight resistance in wheat. *Science*, **368**, eaba5435.

Wang, X. and Wang, L. (2016) GMATA: An Integrated Software Package for Genome-Scale SSR Mining, Marker Development and Viewing. *Front. Plant Sci.*, **7**, 1350.

Xu, Z. and Wang, H. (2007) LTR_FINDER: an efficient tool for the prediction of full-length LTR retrotransposons. *Nucleic Acids Res.*, **35**, W265-268.

Yan, J., Li, X., Wang, L., Li, D., Ji, C., Yang, Z., et al. (2024) A high-continuity and annotated reference genome of allotetraploid Siberian wildrye (*Elymus sibiricus* L., Poaceae: Triticeae). *bioRxiv*, 2024.04.17.589894.

Yang, X., Hu, R., Yin, H., Jenkins, J., Shu, S., Tang, H., et al. (2017) The Kalanchoë genome provides insights into convergent evolution and building blocks of crassulacean acid metabolism. *Nat. Commun.*, **8**, 1899.

Yang, Z. (2007) PAML 4: phylogenetic analysis by maximum likelihood. *Mol. Biol. Evol.*, **24**, 1586–1591.

Zhang, X., Wang, H., Sun, H., Li, Y., Feng, Y., Jiao, C., et al. (2023) A chromosome-scale genome assembly of *Dasypyrum villosum* provides insights into its application

as a broad-spectrum disease resistance resource for wheat improvement. *Mol. Plant*, **16**, 432–451.
